# Epidemiological study and risk factors of equine (*Equus ferus caballus*) gastrointestinal helminth infections in the north and northeast of Iran

**DOI:** 10.1016/j.parepi.2026.e00493

**Published:** 2026-03-19

**Authors:** Faezeh Faghihzadeh Gorji, Soheil Sadr, Hassan Borji

**Affiliations:** Department of Pathobiology, Faculty of Veterinary Medicine, Ferdowsi University of Mashhad, Mashhad, Iran

**Keywords:** Gastrointestinal helminth, Horse, Iran, Epidemiological study, Drug resistance, Risk factors

## Abstract

Understanding the epidemiology of gastrointestinal (GI) helminth infections in equines is critical for investigating drug resistance patterns and developing effective strategies to control and prevent these infections. There is a lack of data regarding horse GI helminths and risk factors in the north and northeast of Iran. Hence, the present study aimed to identify GI helminths in horses in these regions and determine their risk factors. A total of 340 fecal specimens from horses of north (*n* = 141) and northeast (*n* = 199) Iran were coprologically examined for GI helminth infections. The polymerase chain reaction (PCR) technique was used to detect *Strongyloides westeri* (*S. westeri*) eggs in horses. The prevalence of GI helminths in equines was 40.6%, with the highest prevalence reported in horses from the north of Iran at 55.3% (95% CI: 46.8–63.6), compared with 30.2% (95% CI: 23.9–36.9) in horses from northeast Iran. The recovered helminth parasite species were Strongylidae (27.9%; 95/340), *Parascaris* sp. (10%; 34/340), *S. westeri* (3.5%; 12/340), *Oxyuris equi* (1.2%; 4/340), *Anoplocephala perfoliata* (0.6%; 2/340), *Trichuris* spp. (0.3%; 1/340), and *Dicrocoelium dendriticum* (4.7%; 16/340). The coproculture performed on 95 positive *Strongyle* fecal samples revealed *S. vulgaris* at 67.4% (64/95), *S. edentatus* at 29.5% (28/95), Cyathostominae at 49.5% (47/95), and *Trichostrongylus* spp. at 5.3% (5/95). Furthermore, there was a significant association between GI parasite prevalence and feeding type, with free-grazing horses having a higher infection rate than stabled horses. Age was not significantly associated with the overall infection rate. However, only the *Parascaris* sp. infection was significantly higher in younger horses (Group A). Additionally, horses that received anthelminthic treatment had a lower infection rate than those without a history of anthelminthic treatment. The high prevalence of GI helminths in horses in the studied region underscores the need for strategic deworming, broad-spectrum anthelminthics, and a rotational grazing program to control and prevent infections.

## 1 Introduction

Horse racing is one of the most common sports and a leisure activity with great economic importance worldwide (Vamplew, 2020). The horse industry is crucial, with high employment and turnover worldwide (Monterrubio and Pérez, 2021). As an ecotourism attraction, the horse industry can attract many tourists interested in horses and even bring significant foreign exchange revenue to the country (Kalybekov et al., 2019). Challenges facing the horse industry in the Middle East include a lack of attention to the horse's health, environmental issues, and inadequate transportation (Gudeta and Diriba, 2024; Yorke et al., 2017). Furthermore, weak management in public excitement and encouraging people to enter the world of horses, along with a lack of public education about the beneficial aspects of horse use (McGreevy, 2012; Sharpley and Vass, 2006).

Gastrointestinal (GI) helminths can cause weight loss, weakness, colic, and impaired nutrient absorption in horses, and if left untreated, can compromise the horse's overall health (Garber et al., 2020; Ogbein et al., 2022; Raza et al., 2019). Horses are susceptible to several GI helminth infections, and they could simultaneously be infected with different species (Mathewos et al., 2021; Nielsen, 2012; Reinemeyer and Nielsen, 2017; Walshe et al., 2019). Drug resistance in horses is a serious concern that can lead to treatment failure and inadequate infection control (Ahuir-Baraja et al., 2021; Rendle et al., 2024). Drug resistance can be caused by the incorrect and excessive use of anthelminthic administration (Elghryani et al., 2024; McTigue et al., 2022; Sadr et al., 2024).

The most common GI helminths in horses are Strongylinae, Cyathostominae, *Parascaris* sp., *Oxyuris equi (O. equi)*, *Trichostrongylus*, *Dicrocoelium*, *Anoplocephala*, and *Strongyloides westeri* (*S. westeri*) (Kaur et al., 2019; Kuzmina et al., 2005; Nielsen et al., 2010; Saeed et al., 2019). Gastrointestinal helminths significantly cause morbidity and mortality in horses (Craig, 2018; Furtado et al., 2016; Hedberg-Alm et al., 2020). Gastrointestinal helminths can cause mechanical complications, including pressure from their intestinal blockage, and can also compete with the host for food, as seen with the *Ascaris* worm in a foal (Curtis et al., 2019; Mehdi and Mohammad, 2006). In some cases, GI helminths cause severe colic and the death of the horse (Reinemeyer and Nielsen, 2009). Previous studies show a high prevalence and variety of GI helminths in horses in Iran (Hosseini et al., 2009; Hosseinnezhad et al., 2021; Imani-Baran et al., 2019; Sazmand and Joachim, 2017; Tavassoli et al., 2010). Based on available information, gastrointestinal parasites are among the leading causes of equine death in Iran (Borji et al., 2014; Khamesipour et al., 2021; Tavassoli et al., 2016).

Drug resistance among equine intestinal parasites, particularly nematodes, has become a silent crisis in equine health. The frequent and sometimes indiscriminate use of antiparasitic drugs without accurate diagnosis has led to the selection and survival of resistant parasites and perpetuated their life cycle. This phenomenon, which has now become a multi-species problem, means that different classes of drugs, such as benzimidazoles and ivermectin, are losing their effectiveness in controlling populations of these parasites. In such circumstances, the urgent need for accurate, up-to-date epidemiological data is more evident than ever. Gathering information on the prevalence of resistant species across different geographical areas, the pattern of egg shedding across different seasons, and its relationship to the age, breed, and housing conditions of the horse is essential for developing targeted, intelligent treatment protocols.

Understanding the epidemiology of GI helminth infections across regions is imperative for investigating drug resistance patterns and developing effective strategies to control and prevent infections (Matthews et al., 2023). Using epidemiological data, treatment and management programs can be improved, and anthelmintic drug resistance can be prevented. There is a lack of data regarding horse GI helminths and risk factors in the north and northeast of Iran. Hence, the present study aimed to identify GI helminths in horses in these regions and determine their risk factors.

## 2 Methods and materials

### 2.1 Study area

The areas studied were chosen based on their geographical climate from October 2021 to July 2022. Mashhad, the first region investigated, is situated at 36208′ north latitude and 59,380′ east longitude in northeast Iran. At an elevation of 995 m above sea level, Mashhad has a cold climate and receives moderate rainfall each year. It has hot summers, cold winters, and a cold semi-arid climate. Snow is occasionally seen in the city, which receives about 250 mm of precipitation per year. The maximum temperature in summer is 45 °C, and the minimum in winter is −10 °C.

**Fig. 1:**
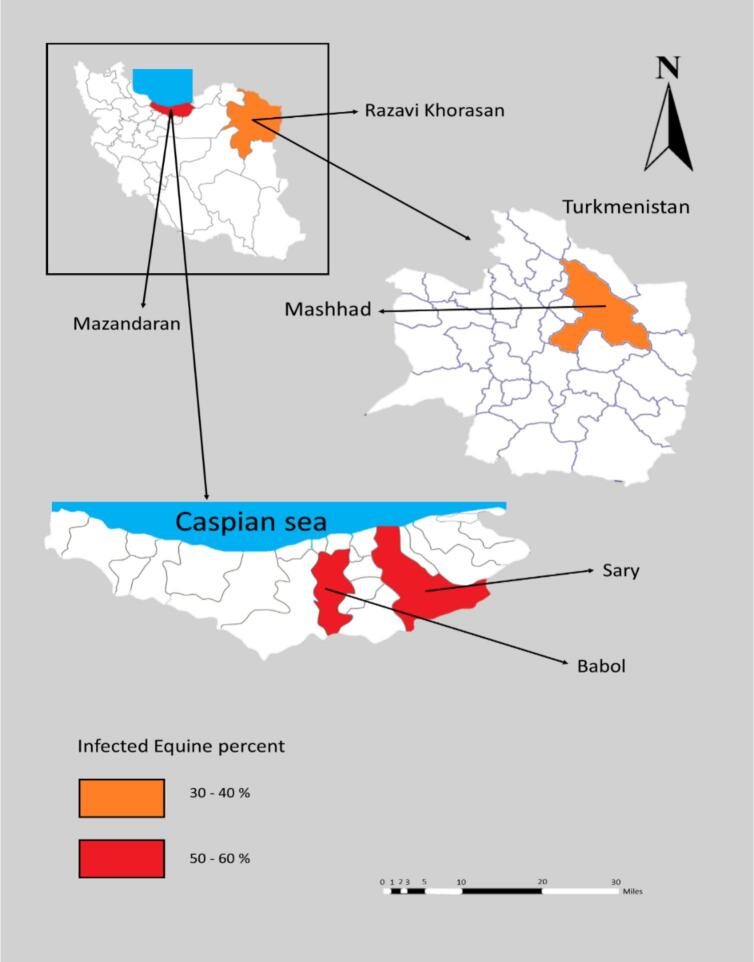
The geographical locations of Mashhad and Babol, where fecal samples were collected from horses for the current study.

The second region, Babol, is situated between the northern slopes of the Alborz Mountains and the southern Caspian Sea. Babol is one of the most important cities in northern Iran, and, due to its location on the west bank of the Babolrud River, which runs approximately 20 km south of the Caspian Sea, the city receives heavy rainfall each year. A tropical subtropical climate with Mediterranean influences dominates Babol (Fig. 1).

### 2.2 Study design

A total of 340 fecal specimens from horses of north (*n* = 141) and northeast (*n* = 199) Iran were coprologically examined for GI helminth infections. Our previous research investigated blood parasites and *Eimeria* infection, but the current study is investigating GI helminths in those horses (Faghihzadeh Gorji et al., 2024a; Faghihzadeh Gorji et al., 2024b; Gorji et al., 2023). The present study was designed using Thrusfield's research to determine the sample size (Khamesipour et al., 2021). In a previous study, a 30% prevalence was used for horses. Hence, Cochran's formula was used to determine the sample size, with a 95% confidence interval and a 5% desired absolute precision. Thus, 340 horses were included in the current study. Of 340 fecal samples, 199 were collected in Mashhad and 141 in Babol. All the horses in this study had birth certificates, and owners were given a questionnaire to obtain detailed information on the horses' characteristics, and the history of anthelminthic administration was also recorded.

The investigated risk factors were age (group A: 1- < 3 years; group B: >3–10 years), sex, type of feeding (stable or free grazing), and history of taking anthelminthic administration.

### 2.3 Sampling, coproculture, and egg counting technique

Fecal specimens were collected directly from the rectum using clean plastic gloves and were transferred to the Veterinary Parasitology Laboratory of Ferdowsi University of Mashhad for further examination. Several techniques were used to examine fecal specimens, including the modified Baermann technique for separation of L3 larvae of small and large *Strongyles*, sedimentation for detection of trematode eggs, and the flotation technique with saturated salt water (1.2 specific gravity) for *Strongyles*. Moreover, coproculture was performed on 10 g of individual fecal specimens cultured at room temperature, separating small strongyle eggs from large ones. For 14 days, fecal specimens were ventilated daily for 1 h and moistened to prevent desiccation. Modified Baermann techniques were used to isolate the third larval stage (L3), and their morphology was determined (Halvarsson et al., 2024; Lichtenfels, 1979; Lichtenfels et al., 2008).

Moreover, the present study used a supersaturated sodium chloride solution as a flotation fluid (Demelash et al., 2016; Zajac et al., 2021), and several keys were used to identify and characterize the helminth eggs (Skryabin, 2023). The number of gut cells, the shape, and the size of the larva's tail were considered to determine the larvae. Based on the number of gut cells observed, different parasitic nematodes were distinguished (Matto et al., 2015; Russell, 1948).

### 2.4 Molecular identification of *Strongyloides westeri*

After detecting *Strongyloides* spp. eggs from infected horses, fresh fecal specimens were collected and sent to the laboratory for analysis. An egg count was performed under a compound light microscope after eggs were collected using the modified McMaster technique. After transferring five ml of the suspension containing nematode eggs to a microcentrifuge tube, the suspension was stored at −20 °C. DNA was extracted and isolated from 250 mL of concentrated egg solution according to the manufacturer's instructions. *Strongyloides* spp. eggs were identified by PCR as described in a previous study (Dorris et al., 2002). In this study, primers SSUA_F (5′-AAAGATTAAGCCATGCATG-3′) and SSU22_R (5′-GCCTGCTGCCTTCCTTGGA-3′) were used for PCR amplification of the partial 5′ variable region (392 base pairs) of 18S DNA. PCR-amplified fragments from purified DNA were visualized on a 2.0% (*w*/*v*) agarose gel stained with cybergreen and visualized with a GelDoc system (Bio-Rad, Hercules, CA).

### 2.5 Statistical analysis

SPSS software version 26 was used to perform statistical analysis. For the statistical analysis of risk factors, we first performed a univariable analysis using the chi-square test. Prevalence estimates for each risk factor category and parasite group were calculated, along with their 95% confidence intervals, using the Wilson score method. To assess the independent contribution of each risk factor while adjusting for potential confounders, we then performed a multivariable logistic regression analysis. The initial model included all five variables of interest (region, anthelmintic history, age, sex, and feed type). Adjusted odds ratios (AORs) and their 95% confidence intervals were derived from this model to quantify the strength of these associations. A *p*-value of less than 0.05 in the multivariable model was considered statistically significant.

### 2.6 Ethical approval

All applicable international, national, and/or institutional guidelines for the care and use of animals were followed, and we confirm that the study was carried out in compliance with the ARRIVE guidelines. The study procedure has been approved by the ethical committee of the Animal Welfare Committee at Ferdowsi University of Mashhad (Approval ID: IR.UM.REC.1400.12656860).

## 3 Results

### 3.1 Prevalence and species

The prevalence of GI helminths in equines was 40.6%, with the highest prevalence reported in horses from the north of Iran at 55.3% (95% CI: 46.8–63.6), compared with 30.2% (95% CI: 23.9–36.9) in horses from northeast Iran. The recovered helminth parasite species were Strongylidae (Strongylinae and Cyathostominae) (27.9%; 95/340), *Parascaris* sp. (10%; 34/340), *S. westeri* (3.5%; 12/340), *Oxyuris equi* (1.2%; 4/340), *Anoplocephala perfoliata* (0.6%; 2/340), *Trichuris* spp. (0.3%; 1/340), and *Dicrocoelium dendriticum* (4.7%; 16/340). Furthermore, the EPG counts in infected horses ranged from 50 to 600 for Strongylidae and from 50 to 180 for *Parascaris* sp. (Table 1).

**Table 1:** Distribution of species of gastrointestinal helminths identified from horses.

Parasite	Number of positive samples	Percent (%)
Strongylidae	95/340	27.9%
*Parascaris* spp	34/340	10%
*Strongyloides westeri*	12/340	3.5%
*Oxyuris equi*	4/340	1.2%
*Trichuris* spp	1/340	0.3%
*Anoplocephala perfoliata*	2/340	0.6%
*Dicrocoelium dendriticum*	16/340	4.7%

The total number of parasite detections (*n* = 164) exceeds the number of infected horses (*n* = 138), indicating that co-infections with multiple parasite species occurred in 18.8% of the positive cases.

### 3.2 Differential larval counts

The coproculture performed on 95 positive *Strongyle* fecal samples revealed *S. vulgaris* at 67.4% (64/95), *S. edentatus* at 29.5% (28/95), Cyathostominae at 49.5% (47/95), and *Trichostrongylus* spp. at 5.3% (5/95) (Table 2).

**Table 2:** Frequency of gastrointestinal helminths in horses after fecal culture.

Parasite	Number of positive samples	Percent (%)
*Strongylus vulgaris*	64/95	67.4%
*Strongylus edentatus*	28/95	29.5%
Cyathostominae	47/95	49.5%
*Trichostrongylus* spp.	5/95	5.3%

### 3.3 Molecular identification of *S. Westeri*

Microscopic examination of 340 fresh fecal samples revealed that 12 fecal samples were infected with small ellipsoidal nematode eggs ranging between 40 and 50 × 50–60 μm, and FECs were 40 and 75 EPG in these samples. PCR amplicons showed a 392 bp band, which was similar to 98.5% of the reference sequence for *S. westeri* (GenBank accession no. AJ417032) (Supplementary Fig. 1).

### 3.4 Risk factors

According to the results, although in the univariable analysis the prevalence of infection was significantly higher in the northern region (55.3% vs. 30.2%), after adjusting for other variables in the logistic regression model, the regional difference was not significant, indicating that this difference is likely due to management factors such as the type of feeding and the use of anthelmintics. Furthermore, there was a significant association between GI parasite prevalence and feeding type, with free-grazing horses having a higher infection rate than stabled horses. Moreover, female horses had a significantly higher infection rate than male horses in the univariable analysis; however, this association was not significant after adjusting for other factors in the multivariable model. Age was not significantly associated with the overall infection rate. However, as expected, only the *Parascaris* sp. infection was significantly higher in younger horses (Group A). Additionally, horses that received anthelminthic treatment, such as Ivermectin and Fenbendazole, had a lower infection rate than those without a history of anthelminthic treatment (Table 3).

**Table 3:** Univariable and multivariable logistic regression analysis of risk factors associated with gastrointestinal helminth infection in horses from north and northeast Iran.

Risk factor	Category	Total (N)	Prevalence % (95% CI)	Crude OR (95% CI)	p-value (Univariable)	Adjusted OR⁎ (95% CI)	p-value (Multivariable)
Region	Northeast	199	30.2% (23.9–36.9)	1.00 (Reference)	< 0.001	1.00 (Reference)	0.251
	North	141	55.3% (46.8–63.6)	2.87 (1.85–4.45)		1.42 (0.78–2.59)	
History of anthelmintics	Yes	269	35.3% (29.7–41.3)	1.00 (Reference)	< 0.001	1.00 (Reference)	0.002
	No	71	63.4% (51.6–74.1)	3.17 (1.85–5.42)		2.68 (1.42–5.06)	
Age group	1–3 years^a^	242	41.3% (35.2–47.7)	1.00 (Reference)	0.932	1.00 (Reference)	0.883
	>3–10 years	98	40.8% (31.3–50.9)	0.98 (0.61–1.57)		1.04 (0.60–1.82)	
Sex	Male	215	33.9% (27.8–40.6)	1.00 (Reference)	< 0.001	1.00 (Reference)	0.104
	Female	125	53.6% (44.7–62.4)	2.25 (1.45–3.50)		1.55 (0.91–2.64)	
Feed type	Stable	299	36.4% (31.1–42.1)	1.00 (Reference)	< 0.001	1.00 (Reference)	0.006
	Free grazing^b^	41	75.6% (60.5–86.7)	5.40 (2.56–11.36)		3.41 (1.42–8.21)	

OR: Odds Ratio; CI: Confidence Interval.

⁎ Adjusted ORs are derived from a multivariable logistic regression model that includes all variables listed in the table (region, anthelmintic history, age, sex, and feed type).

a ***Parascaris* sp. and Age:***Parascaris* sp. infections were predominantly found in younger animals. The prevalence in Group A (1–3 years) was 13.2% (95% CI: 9.3–18.2), which was significantly higher than the 2.0% (95% CI: 0.4–7.3) observed in Group B (>3–10 years). This age distribution is typical, as immunity to *Parascaris* typically develops with age.

b ***Strongyle* spp. and Feed Type:** The multivariable analysis confirmed that free-grazing horses had significantly higher odds of overall GI helminth infection (AOR = 3.27, 95% CI: 1.36–7.86, *p* = 0.008). This risk factor is particularly pronounced for *Strongyle* spp., with a prevalence of 61.0% in free-grazing horses compared to 23.4% in stabled horses. This is likely because free-grazing horses have continuous, more intensive exposure to contaminated pastures, where infective third-stage *strongyle* larvae (L3) accumulate on the herbage, increasing the likelihood of ingestion and subsequent infection.

## 4 Discussion

Gastrointestinal (GI) helminths can cause weight loss, weakness, colic, and impaired nutrient absorption in horses, and if left untreated, can compromise the horse's overall health (Andersen et al., 2013; Chaucheyras-Durand et al., 2022; Nielsen et al., 2023). In the present study, the prevalence of GI helminths in equines was 40.6%, with the highest prevalence reported in horses from the north of Iran at 55.3% (95% CI: 46.8–63.6), compared with 30.2% (95% CI: 23.9–36.9) in horses from northeast Iran, lower than the infection rate in Urmia, northwest Iran (79.2%) (Tavassoli et al., 2010). This difference may be due to the type of feeding, the level of immunity to the GI helminth parasite, the history of anthelminthic administration, and the accessibility to a veterinary clinic.

In the current study, infection prevalence was higher in horses from northern Iran than in horses from northeast Iran. This difference may be due to the regions of the north of Iran being near the Caspian Sea and experiencing humid weather, which provides ideal conditions for the life cycles of GI helminths. In contrast, Mashhad, a dry, hot region with temperatures sometimes reaching 45 °C, is considered an unfavorable environment for the survival and spread of parasitic species. Furthermore, the highest level of contamination was associated with Strongylidae, with 95 cases (27.9%) infected; of these, 63 cases (66.31%) were related to the north and 32 cases (33.68%) to the northeast.

Horse infection with Strongylidae has been reported in different regions of Iran (Alborzi et al., 2020; Ferdowsi et al., 2011) and Thailand (Phetkarl et al., 2024). Horses with Strongylidae are usually infected through contaminated grazing in pastures, and their habitat is in the horse's intestines. Therefore, pasture hygiene and regular anthelminthic administration should be given special attention. A report described anthelmintic resistance in *Strongylids* in wild and domestic equids in Ukraine (Kuzmina et al., 2020).

Similar to the current study, recent research by Adhikari et al. (2024) investigated the prevalence and diversity of GI parasites in working horses in Nepal (Adhikari et al., 2024). Coproscopy was performed using direct wet-mount, formalin-ethyl acetate sedimentation, saturated-salt flotation, and acid-fast staining techniques. The results showed that 90.2% of the fecal specimens were infected, and 15 species were identified, eight of which were zoonotic (*Entamoeba* sp., *Cryptosporidium* sp., *Balantidium coli*, *Blastocystis* sp., *Iodamoeba* sp., *Strongyloides* sp., *Trichostrongylus* sp., and *Fasciola* sp.). The prevalence of parasites was higher in adult horses than in young horses, and simultaneous infection with multiple parasite species was more common than single species. Risk factors such as free grazing, environmental conditions, and poor anthelminthic administration management were significantly associated with increased infection. The results of this study indicate higher parasite species diversity than in our previous research, likely due to the geographical diversity and unique environmental conditions of the studied areas.

*Parascaris* sp. was one of the most prevalent infections, with a 10% infection rate. *Parascaris* sp. infections are more common in stabled horses without access to free pasture, as the closed environment and accumulation of waste in the holding area facilitate this parasite's transmission and life cycle. The current study's findings support this. Infection of horses with *P. equorum* and *P. univalens* has been previously reported from different regions worldwide (Eslami et al., 2014; Nielsen et al., 2014; Sazmand et al., 2020). According to a previous study, ivermectin resistance in *P. univalens* was first reported in Iceland (Martin et al., 2021).

The current study also found a fecal specimen containing *Trichuris* eggs, suggesting that horses may be infected with this parasite; however, further studies are needed to determine the exact *Trichuris* species in horses. Moreover, *Anoplocephala* was found in only two specimens from northern Iran, and this may be related to feeding type, as some horses in the north are free-grazing. Furthermore, the *Dicrocoelium* infection rate was 4.71%, and it has also been reported in Turkey (Soykan and Öge, 2012; Uslu and Guclu, 2007) and Canada (Hazlett et al., 2018). A drug resistance report noted the lack of albendazole efficacy against *D. dendriticum* infection on a sheep farm in France (Petermann et al., 2024).

The factors examined in this study included age, sex, type of nutrition, and history of taking anthelminthic administration. There was no significant relationship between sex and infection rate. Age was not significantly associated with the overall infection rate. However, as expected, only the *Parascaris* sp. infection was significantly higher in younger horses. Furthermore, in studies conducted in Ardabil, Tabriz, Tehran, and Ethiopia, there was no significant association between sex and disease occurrence (Sazmand et al., 2020; Shahbazi et al., 2018). A previous study in Germany found that gender was a factor in *Parascaris* infection (Rehbein et al., 2013). There was also a significant relationship with the factor of a history of taking anthelminthic administration, which was also reported in Ardabil (Shahbazi et al., 2018).

The present study, *S. westeri* was observed in 3.5% of horses in both regions. The modified McMaster fecal egg count technique has been the most commonly used method for diagnosing *S. westeri* (Nápravníková et al., 2019). Similar to the previous study in Australia, the detection of *S. westeri* was molecularly confirmed (Abbas et al., 2021). Furthermore, in 2007, *S. westeri* was identified as the most common parasite by autopsy in Saudi Arabia (Anazi and Alyousif, 2011).

Drug resistance to benzimidazoles (such as fenbendazole) and macrocyclic lactone compounds (such as ivermectin and moxidectin) is a serious and complex issue with wide-ranging effects on equine health and the horse industry's economy (Abbas et al., 2024; Nápravníková et al., 2022; Silva et al., 2019). Improper and excessive use of anthelminthic administration, such as benzimidazoles, macrocyclic lactones, and pyrantel, has led to drug resistance (Cai et al., 2023; Nielsen, 2022; Peña-Espinoza, 2018; Salem et al., 2021). One of the main reasons for drug resistance can be the frequent and indiscriminate use of anthelminthic administration without considering the drug rotation periods. Moreover, incorrect or insufficient drug doses are used, especially in non-clinical conditions or without veterinary supervision. Another factor can be the lack of accurate identification of the parasite type and the use of non-specialized drugs that lack the appropriate effect. This worrying situation poses a serious challenge to the effective management of parasitic infections and underscores the need for a rapid transition from blind, routine treatments to targeted strategies. Therefore, it is recommended that control programs be designed based on fecal egg count tests to identify horses with high excretion (target groups for treatment), prevent excessive drug use, and continuously monitor the effectiveness of drugs used in each area or population by performing fecal egg count reduction tests.

For future studies, it is recommended the use of nanobiosensors that are highly sensitive and rapid diagnostic tools able to combine nanomaterials (gold, graphene, quantum dots) with biological components to detect pathogens (Sadr et al., 2025). Moreover, nanotechnology-based drug delivery systems can effectively reduce drug resistance by improving drug targeting and increasing effective concentrations at the site of infection (Soleymani et al., 2024; Sorouri et al., 2024). Accurate knowledge of the epidemiology of infections in different regions is necessary to overcome drug resistance. This information can help identify drug resistance patterns and design more effective control programs. In addition, improving environmental hygiene can play an essential role in reducing parasite burden. Further research is necessary to develop new anthelminthic administration and more accurate diagnostic methods. Additionally, training veterinarians and horse breeders in the proper administration of anthelmintics and environmental management can help reduce the prevalence and effects of drug resistance.

One limitation of the current study was that it was conducted in only two regions of Iran: Mashhad and Babol. Hence, more studies are needed to compare infection rates across different areas, provide much more detailed information on infection rates in Iran, and compare them with current research. It is suggested that future studies be conducted in other regions of Iran and worldwide to investigate the effects of GI helminths on horse health and their pathogenicity, and to evaluate the efficacy of common anthelmintic drugs.

This is the first comprehensive study in Iran that used multivariate logistic regression analysis to simultaneously examine multiple risk factors, including geographic region, age, sex, type of diet, and history of anthelmintic use, in relation to gastrointestinal worm infections in horses in the north and northeast of the country. The innovation of this study, in addition to the appropriate sample size and providing accurate prevalence of various parasites with a 95% confidence interval, was the specific study of each parasitic group, such as *Strongyloides* and Parascaris, and the identification of independent risk factors affecting them, the results of which can serve as a scientific basis for designing regional and targeted anthelmintic control programs.

## 5 Conclusion

The findings of this study provide a better understanding of the species identification of GI helminths in horse populations in Iran and can also serve as a valuable scientific basis for designing targeted diagnostic, preventive, and control strategies. Given the high prevalence of GI helminth infections in horses, a strategic anthelminthic treatment program is necessary. Given the findings of this study regarding the high prevalence of helminth infections in horses in northern and northeastern Iran and the significant role of factors such as free grazing and lack of use of anthelmintics as the most important risk factors, it seems necessary to conduct additional studies to examine the pattern of anthelmintic resistance, along with designing and implementing regional control programs based on integrated pasture management, educating breeders, and targeted administration of anthelmintics based on microscopic examinations and risk factor assessment, in order to reduce the infection burden, improve the health of the horse population, and prevent the spread of drug resistance.

## CRediT authorship contribution statement

**Faezeh Faghihzadeh Gorji:** Validation, Methodology, Investigation, Formal analysis, Data curation. **Soheil Sadr:** Writing – review & editing, Writing – original draft, Visualization, Validation, Resources, Methodology, Investigation, Formal analysis, Data curation. **Hassan Borji:** Writing – review & editing, Writing – original draft, Visualization, Validation, Supervision, Software, Resources, Project administration, Methodology, Investigation, Funding acquisition, Formal analysis, Data curation, Conceptualization.

## Informed consent statement

Not applicable.

## Ethical approval

All applicable international, national, and/or institutional guidelines for the care and use of animals were followed, and we confirm that the study was carried out in compliance with the ARRIVE guidelines. The study procedure has been approved by the ethical committee of the Animal Welfare Committee at Ferdowsi University of Mashhad (Approval ID: IR.UM.REC.1400.12656860). All the authors have checked Ethical issues (including plagiarism, consent to publish, misconduct, data fabrication and falsification, double publication and submission, and redundancy).

## Funding

10.13039/501100003121Ferdowsi University of Mashhad, Iran funded this research, grant number 56860.

## Declaration of competing interest

The authors declare that they have no known competing financial interests or personal relationships that could have appeared to influence the work reported in this paper.

## Data Availability

The datasets generated during and/or analyzed during the current study are available from the corresponding author upon reasonable request.

## Appendix A Supplementary data

Supplementary material: **Fig. 1**. PCR amplicons showed a 392 bp band, similar to 98.5% of the reference sequence from *S. westeri* (GenBank accession no AJ417032). Image 1
