# Supplementary material for: Epidemiological study and risk factors of equine (Equus ferus caballus) gastrointestinal helminth infections in the north and northeast of Iran
Source: Parasite Epidemiol Control. 2026 Mar 19;33:e00493. doi: 10.1016/j.parepi.2026.e00493 (PMC13059097; doi:10.1016/j.parepi.2026.e00493)
Supplement: Supplementary file 1 — Supplementary material: Fig. 1. PCR amplicons showed a 392 bp band, similar to 98.5% of the reference sequence from S. westeri (GenBank accession no AJ417032). [file mmc1.docx]

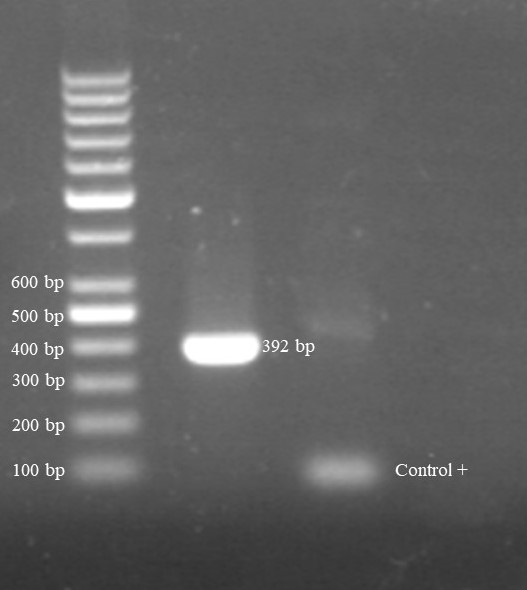


**Supp. Fig. 1**

PCR amplicons showed a 392 bp band, similar to 98.5% of the reference sequence from *S. westeri* (GenBank accession no AJ417032)
